# Predictive Value of Middle Cerebral Artery to Uterine Artery Pulsatility Index Ratio in Hypertensive Disorders of Pregnancy

**DOI:** 10.1155/2015/614747

**Published:** 2015-02-01

**Authors:** Prashanth Adiga, Indumathi Kantharaja, Shripad Hebbar, Lavanya Rai, Shyamala Guruvare, Anjali Mundkur

**Affiliations:** Department of Obstetrics and Gynaecology, Kasturba Medical College, Manipal University, Manipal 576104, India

## Abstract

*Aims and Objectives*. (i) To determine the predictive value of cerebrouterine (CU) ratio (middle cerebral artery to uterine artery pulsatility index, MCA/UT PI) in assessing perinatal outcome among hypertensive disorders of pregnancy. (ii) To compare between CU ratio and CP ratio (MCA/Umbilical artery PI) as a predictor of adverse perinatal outcome. *Methods*. A prospective observational study was done in a tertiary medical college hospital, from September 2012 to August 2013. One hundred singleton pregnancies complicated by hypertension peculiar to pregnancy were enrolled. Both CU and CP ratios were estimated. The perinatal outcomes were studied. *Results*. Both cerebrouterine and cerebroplacental ratios had a better negative predictive value in predicting adverse perinatal outcome. However, both CU and CP ratios when applied together were able to predict adverse outcomes better than individual ratios. The sensitivity, specificity, positive predictive value, and the negative predictive values for an adverse neonatal outcome with CU ratio were 61.3%, 70.3%, 56%, and 78.9%, respectively, compared to 42%, 57.5%, 62%, and 76% as with CP ratio. *Conclusion.* Cerebrouterine ratio and cerebroplacental ratio were complementary to each other in predicting the adverse perinatal outcomes. Individually, both ratios were reassuring for favorable perinatal outcome with high negative predictive value.

## 1. Introduction

Hypertension peculiar to pregnancy (preeclampsia and gestational hypertension) is a pregnancy specific syndrome characterized by reduced organ perfusion secondary to vasospasm and endothelial pathology. There are several hall mark studies which have already established the two arms of the fetal circulation (middle cerebral artery pulsatility index and umbilical artery pulsatility index) both in normal and compromised fetuses. We felt that the actual problem starts from uterine vessels and finally the changes are reflected in the cerebral circulation. We wanted to compare whether alterations in uterocerebral ratio reflect the flow dynamics better than umbilical-cerebral ratio. The vascular changes in these conditions can be reflected in Doppler studies well in advance compared to the conventional antenatal tests of fetal well-being. The brain sparing effect is maximum 2 or 3 weeks before the occurrence of late decelerations on cardiotocogram, suggesting that patient with a high risk for unfavorable pregnancy outcome may have alteration in the blood flow in the middle cerebral artery 2-3 weeks prior to the delivery [1, 2]. As placental insufficiency occurs, several changes occur in fetal circulation, culminating with the brain sparing, characterized by blood flow redistribution with priority to important organs like brain and heart adrenals at the expense of spleen, kidney, and peripheral circulation.

Cerebroplacental (CP) ratio is a well-established predictor of unfavorable pregnancy outcomes, while cerebrouterine (CU) ratio is fairly new ratio of vascular impedance between MCA and uterine arteries, which has not been commonly evaluated [3]. The intent of this study was to know which of the two parameters would help us to predict the perinatal outcome better. Our hypothesis was, therefore, that MCA/uterine artery PI ratio could have a better predictive value for unfavorable outcome than the CP ratio.

## 2. Methodology

This was a prospective observational study, which was carried out over a period from September 2012 to September 2013, in the Department of Obstetrics and Gynecology, in tertiary care center. Eligible participants were those women with singleton pregnancy diagnosed with “hypertension peculiar to pregnancy” (preeclampsia and gestational hypertension), after 26 weeks of gestation. Women with history of chronic hypertension, chronic renal disease, diabetes mellitus, and secondary hypertension due to immunological disease such as SLE and APLA syndrome and women in active labor or those with premature rupture of membranes were excluded. Serial scans by transabdominal route were performed for interval growth and Doppler parameters (umbilical, middle cerebral artery, and uterine artery Doppler). The last Doppler values before the delivery were considered for the study. The biometric parameters were plotted on a customized growth chart to look for any evidence of intrauterine growth restriction. All the ultrasound scans were performed by the first author. Doppler ultrasound was performed as soon as the diagnosis of preeclampsia/gestational hypertension was made. The procedures followed were in accord with the ethical standards of the committee on human experimentation of our institution. Written informed consent was taken from all patients after hospital ethical committee approval.

Preeclampsia was defined as a pregnancy specific syndrome characterized by blood pressure > 140/90 mm of Hg after 20 weeks of gestation with proteinuria > 300 mg/24 hrs or persistent 1+ random dipstick proteinuria and was considered to be severe when associated with blood pressure > 160/110 mm, thrombocytopenia (platelets less than 100,000/*μ*L), renal insufficiency (creatinine greater than 1.1 mg/dL or doubling of baseline), liver involvement (serum transaminases levels twice the normal), cerebral involvement (headache, visual disturbances, persistent nausea or vomiting), or pulmonary edema. Gestational hypertension was defined as blood pressure > 140/90 mm of Hg after 20-week period of gestation with no proteinuria, and blood pressure returns to normal in less than 12 weeks postpartum [4].

Fetal hypoxia was said to exist antenatally, whenever there was absent end diastolic flow or reversal of flow in the umbilical artery, suboptimal NST, and intranatally, when there was thick meconium staining of the amniotic fluid and ominous cardiotocographic changes (persistent and prolonged bradycardia, loss of beat to beat variability, etc.).

Scan was done in recruited patients using Philips HD 11XE machine using 3–5 MHz transabdominal probe. Doppler velocity of uterine artery was recorded at the point at which they crossed over the external iliac artery cranial to crossing of iliac artery. Mean of the PI of both uterine arteries was taken for ratio estimation. The middle cerebral artery was located by color Doppler in a transverse view of fetal brain. The pulsed Doppler sample gate was placed on the vessel about 1 cm of the origin of MCA from the circle of Willis towards lateral edge of orbit. The Umbilical artery PI was obtained from free loop of umbilical cord during fetal apnea. Cerebrouterine ratio (middle cerebral artery to uterine artery PI ratio) and cerebroplacental ratio (middle cerebral artery to umbilical artery PI ratio) were estimated. Cerebrouterine (CU) ratio was plotted on the chart; <5_th_ percentile was considered as decreased or abnormal [3]. Cerebroplacental (CP) ratio was considered as abnormal or to have brain sparing effect, when ratio was <1.08. Patients were followed up till delivery and perinatal outcome was analyzed [3]. The abnormal outcomes studied were small for gestational age, low APGAR, preterm delivery, hyaline membrane disease, assisted ventilation, academia, and overall perinatal outcome.

Table 1 shows the composite score used to calculate the overall perinatal outcome, as more than one adverse outcome was present in many cases. Basic score values of 0, 1, or 2 were assigned to the five outcome variables (birth weight, perinatal death, APGAR at 5 min, respiratory problems, acidemia, and seizure), and the basic score values were summed to obtain an “outcome score” which was called as Modified Tchirikov Composite score for perinatal outcome [5]. This score was constructed after the data had been collected for each new born before further statistical evaluation of overall perinatal outcome. Neonates with scores of more than 2 made up the group of compromised neonates. The data collected was analyzed using Statistical Package for Social Sciences (SPSS, version 16). The validity of the predictive values was analyzed using sensitivity, specificity, and positive and negative predictive values and Chi-square test was used for testing statistical significance. *P* value < 0.05 was considered significant.

Patange and Goel [6] have reported that the cerebroumbilical ratio in normal pregnancy is 1.77 ± 0.43. They noticed that this ratio is reduced to 1.47 (difference of 0.3) when there was placental insufficiency. Based on this information, we calculated sample size with the formula

(1) $$\begin{array}{c} n=\frac{2{\left(z_{1-\alpha /2}+z_{1-\beta }\right)}^{2}}{{\left(\left(\mu_{0}-\mu_{1}\right)/\sigma \right)}^{2}}, \end{array}$$

where *z*_1−*α*/2_ is equal to 1.96 (for *α* = 0.05, i.e., type I error), *z*_1−*β*_ is equal to 0.84 (for *β* = 0.20, i.e., type II error), *μ*_0_ − *μ*_1_ is equal to the difference of means (0.3 as in quoted study), and *σ* is the standard deviation. This equation will give expected power of 0.80. Accordingly, the sample size required is 32 and our sample size of 100 is far more than adequate.

## 3. Results

The patient profile has been described in Table 2. There were a total of 100 cases at initial recruitment (72 primigravidae and 28 multigravidae), gestational hypertension was seen in 64 patients, and 36 had preeclampsia. Out of these 100 cases, 5 cases were lost for followup; thus, perinatal outcome was analysed in 95 patients. Each outcome measure and its relation to cerebrouterine ratio and cerebroplacental ratio were analysed. As prematurity and intrauterine growth restriction can be confounding factors for NICU admission and hyperbilirubinemia; these outcomes were neither correlated with Doppler findings nor were they considered for composite scoring. Out of 95 babies, 48 (50.5%) babies required NICU admission for more than 24 hours. There were 53/95 (55.7%) premature babies of which 34/95 (68%) were below 34 weeks of gestation. Small for gestational age (SGA) neonates were 33/95 (34.7%). Low APGAR (less than 7 at 5 minutes of birth) was seen in 16/95 babies (16.8%), 23/95 babies (24.2%) required assisted respiration, acidemia was present in 10/95 babies (10.5%), hyperbilirubinemia was present in 33/95 (34.7%), and neonatal seizures were seen in only 1/95 (1.05%). Perinatal mortality was present in 5/95 (5.2%) cases of which one was intrauterine fetal death.

When CU and CP ratios were compared, in abnormal CU ratio group, SGA (47.4% versus 26.3%), acidemia (18.4% versus 5.3%), fetal hypoxia (50% versus 22.8%), low APGAR (26.3% versus 10.5%), and adverse perinatal outcome (50% versus 21.1%) were present, which was statistically significant (Table 3). In abnormal CP ratio group, SGA (52.4% versus 29.3%), acidemia (23.8% versus 6.8%), low APGAR (42.7% versus 9.5%), and perinatal outcome (61.9% versus 24.3%) were present, which was also statistically significant. This shows that both of these ratios are fairly accurate in predicting adverse neonatal outcomes. However, CU ratio was better in predicting fetal hypoxia than CP ratio.

Table 4 shows overall performance of CU and CP ratio in predicting perinatal outcome. In the prediction of SGA by CU ratio, the specificity was 67.7%, but negative predictive value (NPV) was higher (73.7%). In prediction of poor APGAR, the NPV of the test was good with 89.5%, whereas the sensitivity and specificity was comparatively lower (i.e., 62.5% and 64.6% resp.). Even for the prediction of the need for assisted ventilation, the NPV of the test was higher (80.7%). In 94.7%, if the cerebrouterine ratio was normal, less likelihood of acidemia was seen. In predicting overall adverse perinatal outcome, specificity was 70.3% and NPV was 78.9%, with low sensitivity and positive predictive value.

In the prediction of SGA with CP ratio, the specificity was higher (83.9%) and NPV was 70.3%. The test had a good specificity of 84.8% and NPV of 90.5%, in ruling out poor APGARs. In prediction of need for assisted respiration, specificity and NPV were higher. In overall prediction of adverse perinatal outcomes, specificity was 87.5% and negative predictive value was 75.7%, indicating that if CP ratio is normal, the likelihood of adverse perinatal outcome is less.

Table 5 shows perinatal outcome for four possible combinations of normal and abnormal CU and CP ratios. It is interesting to note that when both ratios were normal, 76.9% had favorable outcome and when both were abnormal, 81.2% had adverse outcome. Thus it can be inferred that both ratios are complementary to each other in predicting perinatal outcome.

## 4. Discussion

The prevalence of preeclampsia was more in primigravida in our study as is also seen in general. Nulliparous women are at increased risk, which is related to maternal first exposure to chorionic villi [7]. In the present study, abnormal CU ratio was present in 38/95 (40%) cases and abnormal CP ratio (brain sparing) was seen in 21/95 (22%) cases. The mean gestational age of delivery was comparable with that of a study done by as Eser et al. which indicates that gestational age at delivery was significantly lower in group with abnormal CU ratio [8]. When CU ratio was abnormal, SGA was present in 47.4% (18/38) babies and AGA in 52.6% (20/38) babies. However, when CU ratio was normal, 73.7% of the babies were AGA. Thus, with the ratio being normal, the chance of SGA was less likely. Simanaviciute and Gudmundsson found significant correlation with SGA newborn independently with abnormal CP ratio and bilateral uterine artery notching [3]. However, the abnormal CU ratio was not found to be associated with an SGA in the newborn in their study.

The need of an assisted respiration such as continuous positive airway pressure (CPAP) and ventilatory support was studied in relation to CU ratio. Those admitted to NICU with respiratory morbidity were seen in 31.6% (12/38) of babies. When CU ratio was normal, 80.7% (46/57) did not require respiratory assistance. There are no studies available in the current literature which have evaluated the correlation between CU and need for assisted respiration.

Those with normal CU ratio 89.5% (51/57) had good APGARs, indicating that normal CU ratio is reassuring. Simanaviciute and Gudmundsson found no significant correlation between abnormal CU ratio and poor Apgar score [3]. Similar finding was noted in a study by Eser et al. [8].

To study the overall outcome, Modified Tchirikov Composite score for perinatal outcome was used. CU ratio was helpful in ruling out compromised fetus. Preterm delivery was high in those patients with abnormal CU ratio. In cases of severe preeclampsia or imminent eclampsia, the threshold for caesarean delivery was also low. In 11 cases, absent end diastolic flow was noted, of which 2 cases also had severe olighydramnios. Pregnancy was terminated for both maternal and fetal causes, which led to premature delivery in preeclampsia cases. Thus, abnormal CU ratio could not be directly attributed to preterm delivery. In Eser et al. study, CU ratio was independently associated with delivery before 37 weeks, whereas CP ratio was not [8].

Even with severe preeclampsia, some cases had normal CU ratio which can be due to the maternal intake of antihypertensive drug. In a study done by Günenç et al., treatment with methyldopa lowered the uterine artery resistance in preeclamptic patients but did not affect the resistance of umbilical and fetal middle cerebral artery [9]. In two separate studies done by Khalil et al. and Muračević et al., they did not find change in flow resistance of umbilical artery after administration of methyldopa [10, 11].

In our study, 28 patients were on methyldopa and 16 patients were on combination of antihypertensives. CU ratio was abnormal in 17/44 (38.6%) and normal in 27/44 (61.3%). We found that SGA, poor Apgar scores, and preterm delivery rates were higher in the group with abnormal CU ratio than those with normal CU ratio, but poor outcomes were also seen in those with normal ratio, which indicates that there was not much change in fetal hemodynamic changes before and after treatment in cases of severe preeclampsia. Normalization of the Doppler velocimetric indices of the fetal MCA has also been reported in terminal cases [12, 13]. This could have also resulted in normal CU ratio in patients with severe preeclampsia, although the pathological changes would have already occurred.

## 5. Conclusion

The current study found that in the diagnosis of the complications of hypertension peculiar to pregnancy (preeclampsia and gestational hypertension), CU ratio and CP ratio were complimentary to each other in predicting adverse perinatal outcomes than the independent ratios alone.

## Figures and Tables

**Table 1:** Modified Tchirikov Composite score for perinatal outcome.

Outcome	0	1	2
Birth weight	>90th centile	10th–90th centile	<10th centile
Perinatal death	Absent	—	Present
APGAR	>7	5–7	<5
Respiratory problems	Grunting	Ventilator support	Hyaline membrane disease
Acidemia	>7.2	7.1-7.2	<7.2
Seizure	Absent	—	Present

>/=2: unfavourable score, <2: favourable score.

**Table 2:** Patient profile (*n* = 95).

Maternal age in years	28.45 ± 4.665 (20–41)
Gestation in weeks at examination	33.82 ± 3.473 (26–39)
Gestation in weeks at delivery	35.47 ± 2.752 (27–40)
Uterine artery pulsatility index	1.02 ± 0.496 (0.35–2.7)
Middle cerebral artery pulsatility index	1.52 ± 0.661 (0.6–6)
Umbilical artery pulsatility index	1.09 ± 0.582 (0.30–3.7)

Values are given as mean ± standard deviation (range).

**Table 3:** Comparison between CP and CU ratios in predicting perinatal outcomes.

Outcome	CU ratio			CP ratio		
	Abnormal	Normal	*P* value	Abnormal	Normal	*P* value
	*n* (%)	*n* (%)		*n* (%)	*n* (%)	
SGA	18 (47.4)	15 (26.3)	**0.035**	11 (52.4)	22 (29.3)	**0.054**
Acidemia	7 (18.4)	3 (5.3)	**0.041**	5 (23.8)	5 (6.80)	**0.025**
Fetal hypoxia	19 (50)	13 (22.8)	**0.006**	9 (42.9)	23 (31.1)	0.314
Low Apgar	10 (26.3)	6 (10.5)	**0.044**	9 (42.7)	7 (9.5)	**0.000**
HMD	6 (15.8)	5 (8.8)	0.295	5 (23.8)	6 (8.1)	**0.025**
Assisted respiration	12 (31.6)	11 (19.3)	0.171	7 (33.3)	16 (21.6)	0.269
Perinatal outcome	19 (50)	12 (21.1)	**0.003**	13 (61.9)	18 (24.3)	**0.001**

*P* < 0.05 significant.

**Table 4:** Overall performance of CU and CP ratios in predicting perinatal outcome (based on Modified Tchirikov Composite score).

Outcome	CU ratio					CP ratio				
	Sensitivity	Specificity	PPV	NPV	Accuracy	Sensitivity	Specificity	PPV	NPV	Accuracy
	(%)	(%)	(%)	(%)	(%)	(%)	(%)	(%)	(%)	(%)
SGA	54.5	67.7	47.4	73.7	63.2	33.3	83.9	52.4	70.3	66.3
Poor Apgar	62.5	64.6	26.2	89.5	64.2	56.3	84.8	42.9	90.5	80.0
Assisted respiration	52.2	63.9	31.6	80.7	61.1	30.4	80.6	33.3	78.4	68.9
Acidemia	70	63.5	18.4	94.7	64.2	50	81.2	23.8	93.2	77.9
Adverse perinatal outcome	61.3	70.3	50.0	78.9	67.4	41.9	87.5	61.9	75.7	72.6

**Table 5:** Perinatal outcome in relation to both CU and CP ratios (based on Modified Tchirikov Composite score).

Perinatal outcome	CU and CP ratio			
	Both ratios normal	Only CU ratio abnormal	Only CP ratio abnormal	Both ratios abnormal
	*n* (%)	*n* (%)	*n* (%)	*n* (%)
Adverse outcome *n* = 31	12 (23.1)	6 (27.3)	0	13 (81.2)
Favourable outcome *n* = 64	40 (76.9)	16 (72.7)	5 (100)	3 (18.8)

## Strength and Limitations of the Study

The strength of the study is the adequacy of the sample size. There are few studies in the past which have studied the comparison of these parameters. However, the limitation of this study is the confounding factors like the use of antihypertensive agents and gestational age for more than 37 weeks. We know that the addition of antihypertensives may to some extent bring about resistance changes in the uterine vessel. We are also aware that beyond 37 weeks, the placenta tries to compensate for the placental insufficiency by remodeling itself.
